# PRPI-SC: an ensemble deep learning model for predicting plant lncRNA-protein interactions

**DOI:** 10.1186/s12859-021-04328-9

**Published:** 2021-08-24

**Authors:** Haoran Zhou, Jael Sanyanda Wekesa, Yushi Luan, Jun Meng

**Affiliations:** 1 School of Computer Science and Technology, Dalian University of Technology, Dalian, 116024 Liaoning China; 2 School of Bioengineering, Dalian University of Technology, Dalian, 116024 Liaoning China

**Keywords:** lncRNA-protein, k-Mer, Stacked denoising autoencoder, Convolutional neural network

## Abstract

**Background:**

Plant long non-coding RNAs (lncRNAs) play vital roles in many biological processes mainly through interactions with RNA-binding protein (RBP). To understand the function of lncRNAs, a fundamental method is to identify which types of proteins interact with the lncRNAs. However, the models or rules of interactions are a major challenge when calculating and estimating the types of RBP.

**Results:**

In this study, we propose an ensemble deep learning model to predict plant lncRNA-protein interactions using stacked denoising autoencoder and convolutional neural network based on sequence and structural information, named PRPI-SC. PRPI-SC predicts interactions between lncRNAs and proteins based on the k-mer features of RNAs and proteins. Experiments proved good results on *Arabidopsis thaliana* and *Zea mays* datasets (ATH948 and ZEA22133). The accuracy rates of ATH948 and ZEA22133 datasets were 88.9% and 82.6%, respectively. PRPI-SC also performed well on some public RNA protein interaction datasets.

**Conclusions:**

PRPI-SC accurately predicts the interaction between plant lncRNA and protein, which plays a guiding role in studying the function and expression of plant lncRNA. At the same time, PRPI-SC has a strong generalization ability and good prediction effect for non-plant data.

## Background

Long non-coding RNA (lncRNA) is a type of RNA molecule with special functions in eukaryotic cells [1]. lncRNA are non-protein coding transcripts and populous with the length of more than 200nt. They extensively exist in the nucleus or cytoplasm. Researchers have found that lncRNAs are involved in regulating multiple crucial biological processes by interacting with protein like chromatin-modified complexes and transcription factors [2–4]. The interactions are relevant to the vital activities of organisms [5–8]. Many key cellular processes, such as signal transduction, chromosome replication, material transport, mitosis, transcription, and translation, are all linked to the interactions between lncRNAs and proteins [9–11]. Although the regulatory role of lncRNAs on gene expression is undisputed, few studies have been done on the function and mechanisms of lncRNAs. Since the regulatory performance of lncRNAs requires the coordination of protein molecules, it is necessary to identify the interactions between lncRNAs and protein molecules.

Most of the research work focuses on the interaction between lncRNA and protein of humans and animals, but less on plants. Compared with animals and humans, the homology of plant RNA is poor. Regulation of gene expression at the post-transcriptional level is mainly achieved by proteins containing well-defined sequence motifs involved in RNA binding. The most widely spread motifs are the RNA recognition motif (RRM) and the K homology (KH) domain. The Arabidopsis genome encodes 196 RRM-containing proteins, a more complex set than found in Caenorhabditis elegans and Drosophila melanogaster. In addition, the Arabidopsis genome contains 26 KH domain proteins. Most of the Arabidopsis RRM-containing proteins can be classified into structural and/or functional groups, based on similarity with either known metazoan or Arabidopsis proteins. Approximately 50% of Arabidopsis RRM-containing proteins do not have obvious homologs in metazoa, and for most of those that are predicted to be orthologous of metazoan proteins, no experimental data exist to confirm this. Additionally, the function of most Arabidopsis RRM proteins and all KH proteins is unknown. However, the higher complexity of RNA-binding proteins in Arabidopsis may account for the observed differences in mRNA maturation between plants and metazoa [12].

There are many lncRNA databases available, but most are focused on humans and vertebrates. Databases from plants include: NONCODE [13], PNRD database [14], PLncDB database [15]. These lcnRNAs play significant roles in guiding reproductive development, growth, stress response, chromosome modification, and protein interactions.

Interactions between lncRNAs and proteins are ubiquitous. Only a few conventional methods such as X-ray diffraction [16], nuclear magnetic resonance [17], electron microscopy [18], neutron scattering [19], cross-linking immunoprecipitation [20] and miRNAs as mediators in a heterogeneous network [21] have been used to detect structural data of protein complexes. This is due to the shortcomings of experiments, like high cost, long time, and complicated test process. Advanced high-throughput sequencing technology has enabled researchers to quickly acquire mass transcriptome and proteomic information, including RNA protein interaction (RPI) real-time analysis. However, conventional experiments have their limits such as they are only used for specific proteins, RNAs, or protein-RNA complexes. Therefore, machine learning has extensively been applied to bioinformatics, such as making multi-labels classification and disease prediction based on given lncRNAs [22] and identifying RNA pseudouridine sites [23]. Muppirala et al. [24] put forward RPISeq, which feeds the sequence coding vectors of RNA and protein by conjoint triad feature (CTF) [25] to the random forest (RF) and support vector machine (SVM) to make predictions. Lu et al. [26] create a method named lncPro, which is based on the fisher linear discriminant approach and uses secondary structure, hydrogen-bond, and van der Waals propensities as input features. IPMiner use the stacked auto-encoder (SAE) and predicts the RNA–protein interactions by RF classifier [27]. Yi et al. [28] propose the RPI-SAN model by using the deep-learning stacked auto-encoder network to mine the hidden high-level features from RNA and protein sequences and feed them into a RF model to predict ncRNA binding proteins. Traditional machine learning methods extract features manually, such as building and extracting features according to physical and chemical characteristics or biological functions. The quality of feature selection directly affects the performance of model prediction. The deep learning method only needs to select the appropriate coding method without building features, so it is more applicable.

Since researchers have to collect features manually through traditional machine learning models, they are not likely to accurately position hidden relationships among the raw data. Nevertheless, deep learning provides a solution. With a multi-layer neural network model architecture [29–31], deep learning enables the automatic extraction of abstract features from datasets. Deep learning has outperformed other commonly used machine learning approaches in image analysis [32], speech recognition, and signal processing [33]. It has also been widely applied in bioinformatics [34, 35]. For example, deep learning has been successfully applied to predict splicing patterns [36], discrimination of breast cancer with microcalcifications on mammography [37] and protein interaction network reconstruction [38]. Compared with other sequence methods, deep learning automatically learns the sequence features of RNAs and protein molecules, discovers specific correlations among the sequences [39], and suppresses noises on the original data by learning the actual hidden advanced features. Besides, with the artificial introduction of noises to some deep learning models, over-fitting is decreased, the generalization ability and robustness of such models are improved.

Ensemble learning is considered the state-of-the-art solution for many machine-learning challenges [40, 41]. Such methods improve the predictive performance of a single model by training multiple models and combining their predictions. Ensemble learning is also widely used in the field of bioinformatics, such as the prediction of miRNA-Disease Association [42].

**Fig. 1:**
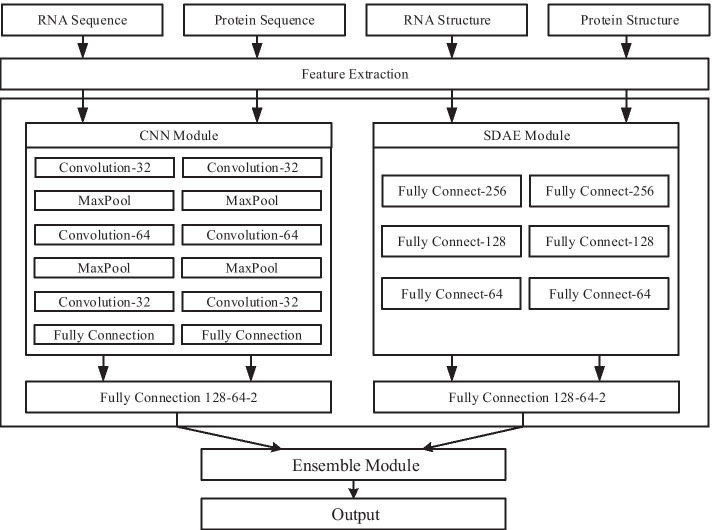
The flowchart of PRPI-SC

In this paper, we proposed a sequence- and structure-based ensemble model for predicting plant lncRNA-protein interaction using stacked denoising autoencoder (SDAE) and convolutional neural network (CNN), named PRPI-SC. The architecture is shown in Fig. 1. The sequence and structure features were extracted from lncRNAs and proteins [23]. Based on the physicochemical properties of protein molecules, 20 protein amino acids were divided into 7 groups [43], embedded into a matrix, and extracted features using SDAE and CNN. After these two modules complete the prediction, the results are integrated and the final results are obtained. The performance of PRPI-SC was tested on plant datasets and other common RNA–protein datasets compared with other methods. The results show that PRPI-SC has excellent performance on plant datasets, and has achieved the best results in accuracy and other evaluation metrics. PRPI-SC effectively predicts the interaction between plant lncRNA and protein. Experiments on public datasets show that it has good generalization ability and strong robustness.

## Results

### Effect of structural information

To explore whether the added secondary structure information has a positive effect on the final results of the model, we conducted experiments on the ATH948 dataset. The results are shown in Table 1.

**Table 1:** Comparisons of whether to add structure information on dataset ATH948 (%)

Dataset	Input data	Acc	Pre	Sn	Sp	MCC	AUC
ATH948	Only Sequence	88.8	91.2	84.3	91.1	78.1	94.8
	Sequence and Structure	88.9	91.4	84.2	91.8	78.1	95.0

According to the experimental results, accuracy, precision and specificity are increased by 0.1%, 0.2%, and 0.7% respectively after adding secondary structure information, which proves that the structure information can supplement the sequence information and improve the prediction performance of the model.

### Performance comparison between different modules of PRPI-SC

**Fig. 2:**
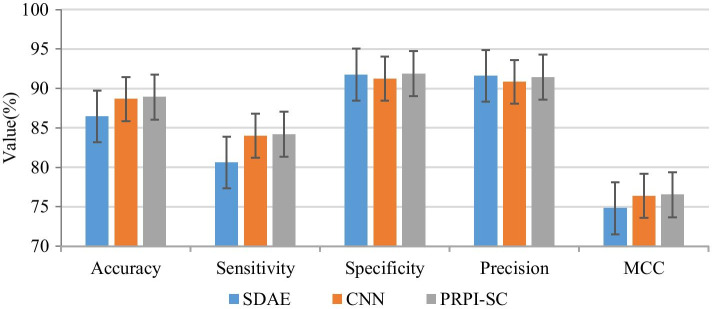
Performance comparison among different prediction modules

PRPI-SC combines two basic prediction modules, SDAE and CNN. We compared each module on the dataset ATH948, and the results are shown in Fig. 2. CNN and SDAE had their advantages in different indicators, but the ensemble module, PRPI-SC is better than a single module. It is showed that our ensemble strategy is effective.

### Performance comparison on plant lncRNA-protein datasets

**Fig. 3:**
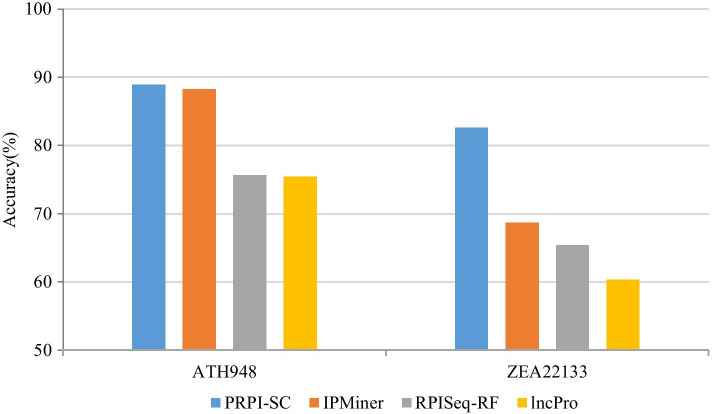
Accuracy comparison among different methods on datasets ATH948 and ZEA22133

We compared PRPI-SC with other RPI prediction methods, such as IPMiner, RPISeq and lncPro, on our datasets, and the accuracies are shown in Fig. 3. In [24], the authors proposed RPISeq-RF and RPISeq-SVM for predicting RNA–protein interaction, and RPISeq-RF performed better than RPISeq-SVM on most datasets. Accordingly, here we only compared PRPI-SC with RPISeq-RF. PRPI-SC achieved good results on our two plant datasets. It achieved the best results on the accuracy, precision and specificity and the second-best result in sensitivity. On the ZEA22133 data set, the accuracy of PRPI-SC was 13.9% higher than IPMiner, which was a great improvement and reaches 99.9% in precision and specificity. Based on the synthetic results, PRPI-SC predicted the interaction of plant lncRNA-protein well, with high accuracy, which was ahead of other RPI prediction methods. Detailed results are shown in Table 2.

**Table 2:** Performance comparison among different methods on datasets ATH948 and ZEA22133 (%)

Dataset	Method	Acc	Pre	Sn	Sp	MCC	AUC
ATH948	PRPI-SC	**88.9**	**91.4**	84.2	**91.8**	78.1	**95.0**
	IPMiner	88.2	89.2	**86.9**	89.5	76.5	94.1
	RPISeq-RF	75.6	76.2	75.2	73.0	**79.4**	90.2
	lncPro	75.4	76.9	75.4	74.7	71.5	89.2
ZEA22133	PRPI-SC	**82.6**	**99.9**	65.2	**99.9**	**69.6**	**92.7**
	IPMiner	68.7	69.6	**66.5**	70.9	37.5	84.6
	RPISeq-RF	65.4	64.1	62.5	70.3	35.9	81.4
	lncPro	60.3	61.3	60.8	69.6	30.9	80.8

The best results are highlighted in bold

### Performance comparison on other published RNA–protein datasets

**Fig. 4:**
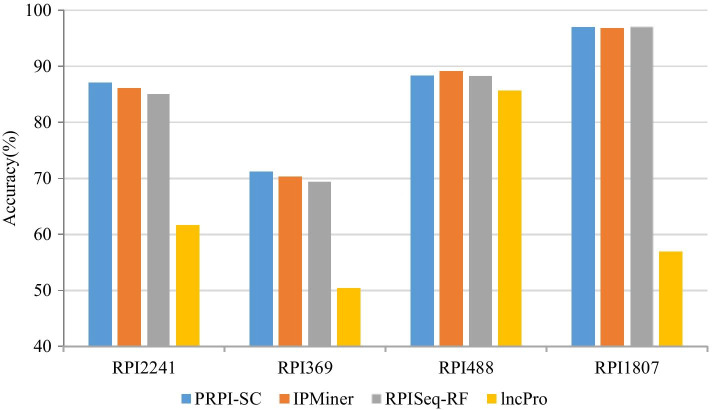
Accuracy comparison among different methods on public datasets

To test the robustness of PRPI-SC, we compared it with other RPI prediction methods on other published RNA–protein datasets and the accuracies are shown in Fig. 4. On the RPI2241 and RPI369 datasets, PRPI-SC achieved the highest accuracy, sensitivity and MCC, and the second best in other performance indexes. On the RPI1807 dataset, PRPI-SC achieved the highest accuracy of 97.0% and the highest MCC of 93.8%, similar to RPISeq-RF method. The performance of RPI488 dataset was relatively average, but the performance indexes are not significantly different from other methods. This is understandable because no prediction method or deep learning model can handle all prediction problems or adapt to all data sets. Detailed results of the performance indexes are shown in Table 3.

**Table 3:** Performance comparison among different methods on public datasets (%)

Dataset	Method	Acc	Pre	Sn	Sp	MCC	AUC
RPI2241	PRPI-SC	**87.1**	85.2	**89.2**	**85.8**	**74.3**	**94.6**
	IPMiner	86.1	**88.2**	87.7	84.1	72.4	90.6
	RPISeq-RF	85.0	86.3	86.1	83.8	70.7	69.0
	lncPro	61.6	66.9	52.9	69.5	31.0	72.2
RPI369	PRPI-SC	**71.2**	66.1	**76.4**	69.6	**42.9**	**80.4**
	IPMiner	70.3	**72.4**	72.3	**72.3**	42.8	77.3
	RPISeq-RF	69.4	70.7	70.5	70.2	40.6	76.7
	lncPro	50.4	71.3	70.8	69.6	40.9	74.0
RPI488	RPPI-SC	88.3	92.2	**84.3**	91.8	77.1	90.5
	IPMiner	**89.1**	93.5	84.0	**94.4**	**78.8**	91.4
	RPISeq-RF	88.3	93.5	82.8	83.6	77.2	88.3
	lncPro	85.6	**94.1**	77.6	94.0	72.5	**92.9**
RPI1807	PRPI-SC	**97.0**	95.7	**97.9**	96.6	**93.8**	99.3
	IPMiner	96.8	95.5	96.5	96.5	93.5	**99.8**
	RPISeq-RF	**97.0**	**96.2**	97.0	**97.6**	**93.8**	99.6
	lncPro	56.9	55.5	56.5	58.1	43.8	99.4

The best results are highlighted in bold

## Discussion

The ensemble deep learning model PRPI-SC takes advantage of two different prediction modules, and gives more comprehensive prediction results. CNN architecture has a more powerful fitting ability for k-mer features of sequence and structural information of RNA and Protein and extracts advanced features better. Compared to SDAE-based architecture, CNN architecture performs better in advanced feature representation. SDAE has strong noise reduction capabilities, which can effectively eliminate the interference from noise data, which is more common in plant datasets. Compared with previous methods, PRPI-SC shows good performance in predicting plant RPI.

When training deep learning neural networks, we usually hope to get the best generalization performance that fits the data well. However, all the deep learning neural network structures are prone to overfitting. When the network performance in the training set performs better and the error rate is getting lower and lower, at some point its performance in the test set begins to deteriorate. The generalization ability of a model is usually evaluated by the performance of the model on the validation set. When the model performs well on the training set and poorly on the validation set, we think that the model has overfitting.

To reduce overfitting, the early stopping method is widely used. It calculates the performance of the model on the verification set during training. When the performance of the model on the verification set begins to decline, stop the training to avoid the overfitting problem. To further reduce the impact of overfitting, we set dropout to 0.5 [44].

Compared with the deep learning models dealing with other problems (image recognition, text processing, etc.), our RPI datasets are relatively small in size, except ZEA22133, which is a disadvantage for the deep learning model. In addition to the small amount of data, the selection of negative pairs is also a question worthy of consideration. In ATH948, ZEA22133, RPI369, and RPI2241, negative pairs are generated by random matching after excluding positive pairs, which may cause uneven distribution of data on negative pairs and affect the final results. In our future work, we will also focus on how to optimize the model for small sample size datasets and how to generate more reasonable negative pairs.

## Conclusions

In this study, we propose an ensemble deep learning model PRPI-SC, to input the sequence and structural information of encoded RNA and protein, and to generate comprehensive prediction results using deep learning modules such as SDAE and CNN.

After adding structural information, the overall performance of the model was improved, which shows that secondary structural information play a complementary role to sequence information and helps to improve the prediction results of RPI problems.

PRPI-SC performs very well on plant datasets and is superior to other methods in most performance indicators such as accuracy. In the ZEA22133 dataset, the accuracy is improved by 13.9%. This shows that PRPI-SC can effectively predict the RPI interaction of plants and achieve the expected results. *Arabidopsis* is the representative of dicotyledons, and *Zea mays* is the representative of monocotyledons. This model has a good effect on *Arabidopsis* and *Zea mays* data set, which shows that it can be further extended to other plant data. PRPI-SC also shows good prediction ability on RPI datasets of other mixture species, which indicates that it has good generalization ability and can meet different needs.

## Methods

### Datasets

We created two lncRNA-protein interactions datasets, ATH948 and ZEA22133, representing *Arabidopsis thaliana* and *Zea mays*, respectively. Firstly, we downloaded data from PlncRNADB [45] and used the CD-HIT [46] tool to eliminate redundant sequences with sequence similarity of more than 90% for both protein and lncRNA sequences, thus reducing sequence similarity and experimental bias. Since there are no non-interaction pairs validated by biological experiments, we randomly select the same number of negative pairs in the remaining data by pairing proteins with lncRNAs and removing the existing positive pairs [23]. Using this method, we obtained ATH948 datasets consisting of 35 protein chains and 109 lncRNA chains, including 948 interactive pairs and 948 non-interactive pairs. Similarly, we obtained the ZEA22133 dataset consisting of 42 protein chains and 1704 lncRNA chains, including 22133 pairs of interactive pairs and 22123 pairs of non-interactive pairs. Because of the poor homology of plant lncRNA, we cannot mix different kinds of plant data, to avoid the deep learning model from extracting wrong features which affect the prediction accuracy. We found that the two datasets contain minor lncRNA and protein chains, but they produce a large number of interaction pairs, which may cause noise and increase the difficulty of feature extraction. The details are shown in Table 4.

**Table 4:** Experimental datasets

Dataset	lncRNA	Protein	Interaction pair	Non-interaction pair
ATH948	109	35	948	948
ZEA22133	1704	42	22133	22133
RPI2241	842	2043	2241	2241
RPI369	332	338	369	369
RPI488	25	247	243	245
RPI1807	1078	1807	1807	1436

To test the robustness of PRPI-SC, we collected other RNA–protein datasets from previous studies, such as RPI1807 [47], RPI369 [23], RPI2241 [23], and RPI488 [27]. These four datasets are constructed according to the minimum atomic distance criterion such that if the distance between protein atoms and RNA atoms is less than the specified distance threshold, then protein and RNA pair is considered to be interaction pairs. They are all made up of a mixture of multi-species RNA–protein samples, including animals, plants and humans, and the length of RNA samples varies.

We use different methods to predict the structural information of RNA and protein. For RNA, we use the RNAfold program in ViennaRNA Package [48] to calculate secondary structure information of RNA with minimum free energy, which can be expressed by “.” and “()”. For protein, we use network server SOPMA [49] to predict the structure. The protein sequence is uploaded, and classical trimorphic structure is predicted, including *α*-helix, *β*-sheet and coil.

### Sequence information processing

RNA and protein sequences cannot be directly used as input in deep learning models, thus, proper sequence coding methods have a great impact on the performance of the model. Because the length of RNA and protein sequences in datasets varies widely (20–3000), some common digital matrix coding methods (such as one-hot encoding) are not suitable for RNA and protein sequences, which make the matrix too large and sparse. Therefore, we used k-mer [27] to encode the input sequence and structure information to ensure that the length of the generated digital vector was consistent.

For RNA sequences, the usual method is to extract the 4-mer frequency features of RNA sequences (each sequence consists of A, C, G, T) to obtain 4*4*4*4 = 256 dimensional features. Each eigenvalue is the normalized frequency of 4-mer nucleotides in the RNA sequence, namely AAAA…CATC…TTTT. To fully extract the sequence features of RNA, we added 1-3mer features to form a total of 340-dimensional features. For protein sequences, existing studies have shown that binding residues are more likely to form amino acids with certain properties. Based on the physicochemical properties and interaction of amino acids, 20 kinds of amino acids were classified into 7 categories. They include {Val, Gly, Ala} {Phe, Pro, Leu, Ile} {Ser, Tyr, Met, Thr} {His, Asn, Tpr, Gln} {Arg, Lys} {Glu, Asp} and {Cys}. According to the above rules, we divided the protein sequence into seven groups, extract the 3-mer features of protein trimer, and obtained the 7*7*7 = 343 dimensional features. Similarly, we complemented the 1-2mer feature to form 399 dimensional features. If the k-value of k-mer feature extraction method becomes larger, it will lead to too many zeros in the feature vector and affect the prediction model impact. This is also the method adopted by most articles.

For the processing of structural information, we adopted a calculation method similar to sequence information, which was added to model input as supplementary information. For protein structure, we extracted 1–3 polymer frequencies (*α*-helix, *β*-sheet and coil) of secondary structure to obtain 39-dimensional features; for RNA structure, we extracted 1–4 polymer frequencies (points and scaffolds) of secondary structure to obtain 30-dimensional features. The features of these secondary structure information were integrated with those extracted from previous sequence information to obtain the protein-coding vectors of 438-dimensional features and RNA coding vectors of 370-dimensional features.

### Stacked denoising autoencoder

Autoencoder (AE) belongs to unsupervised learning and does not need labeled training samples. When an autoencoder learns input samples, its training objective is to reconstruct the input signal from the target expression. Therefore, the output is often set to the input itself in training. AE structure can be divided into two parts: encoder and decoder. The encoder maps the transformation from input vector *x* to output representation *y*. The typical expression is:

1 $$y = s\left( {Wx + b} \right)$$

where *s* is a non-linear function, such as sigmoid. *W* is the link weight from the input layer to the middle layer, and *b* is the bias of the middle layer. The decoder maps the output representation *y* back to the input space and reconstructs the vector *z*. The typical form is:

2 $$z = s\left( {W^{{\prime }} y + b^{{\prime }} } \right)$$

where *s* is a non-linear function, such as sigmoid. *W*' is the link weight from the middle layer to the output layer, *b*' is the bias of the output layer, and *z* is regarded as the prediction of *x*. In general, *z* is not an accurate reconstruction of the input variable *x*, it can only approach *x* to the greatest extent.

Denoising autoencoders (DAE) have the same structure as traditional AE, but noise is added to the sample input. Its learning goal is to reconstruct the pure input from the polluted input. The purpose is to filter the noise in the input data, to avoid the occurrence of over-fitting to enhance the generalization ability of the model.

**Fig. 5:**
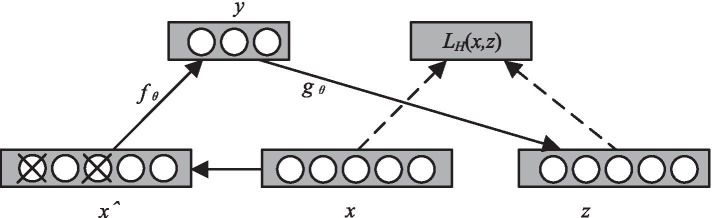
The flowchart of denoising autoencoder

As shown in Fig. 5, unlike traditional AE, signal *y* is reconstructed from noise-contaminated signal *x*ˆ. In general, there are two ways to add noise: one is to add Gaussian noise with the same distribution as the input data, and the other is to set the component of the input vector to 0 with a certain probability. By calculating *y* and *z* with the corrupted data *x*ˆ and iterating errors with *z* and the original *x*, the network learns the corrupted data. Each time sample *x* is trained, a different *x*ˆ is generated.

**Fig. 6:**
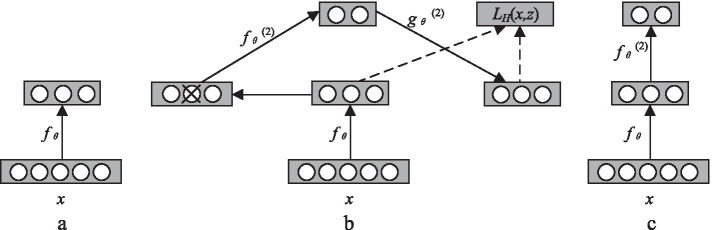
The calculation process of SDAE. **a** Training process of the first layer of DAE. **b** Output of the first layer serves as the input of the second layer. **c** Repeated training in multi-layer deep network

To obtain more advanced feature representation, the DAE is stacked layer by layer in the form of deep network structure to form a model structure that is connected by the DAE top and bottom, namely SDAE [50]. During training, the output of the former layer acts as the pure input of the latter layer, and the training is carried out layer by layer. The learning process is shown in Fig. 6.

Figure 6a shows the first layer of DAE. The function *f*_*θ*_ is used to denoise the input *x*. Figure 6b shows that the output of the first layer is input as the sample of the second layer, and the coding function *f*_*θ*_^(2)^ of the second layer is trained. The training process of the whole deep network is repeated, as shown in Fig. 6c.

### Model design

We designed a deep learning framework, PRPI-SC, to address plant lncRNA-protein interaction problems. After the encoding portion, the CNN and SDAE extract features from the input and form a high-level representation. Finally, the ensemble module integrates the outputs of the two basic modules to form the overall structure of the PRPI-SC.

In the CNN module, two similar sequence-embedding levels were first formed by analyzing the RNA and protein input vectors by CNN, respectively. Then, a three-layer fully-connected part embeds the two sequences as input and performs cross-predictions. There are three convolution layers in each sequence embedding part. Between the two convolutional layers, the max-pooling layer was used to reduce the representation dimension and introduce noise invariance. After the last convolutional layer, the two-dimensional tensor of output was flattened and further used as an input to the fully connected layer. Then, two sequences of RNA and protein were embedded in the representation. Finally, the output of the last layer was the predicted result, which is further integrated by the later ensemble modules.

In the SDAE module, RNA and protein input vectors were first sequenced separately using SDAE to generate two sequence embedding layers. Then, the three-layer fully-connected part concatenated the two sequences as inputs and performed cross-predictions. Through the dimensionality reduction and high-level feature extraction of two three-layer SDAE parts, the sequence embedding representation of RNA and protein was obtained. Finally, a three-layer fully-connected part inserted the first two sequences together as input to its first layer and predicted interactions for specific RNA–protein pairs in the third layer.

The final ensemble module linked the predictions of the CNN module and the SDAE module as the input tensors and produced a more comprehensive prediction for a given lncRNA-protein pair. The two basic modules and ensemble modules use the softmax activation function at their last layers to make binary predictions and use the back-propagation algorithm to minimize loss function of binary cross-entropy. Two optimization methods, Adam and stochastic gradient descent (SGD) are employed successively to train each module, among which Adam first gives the module a quick converge and then SGD is used to fine-tune the module after. During the unsupervised pre-training process of the three-layer SAE, its parameters are optimized by greedy layer-wise training. To avoid the over-fitting problem, the techniques of dropout and early stopping are also used.

### Evaluation of model performance

In this study, we classify protein and lncRNA pairs as interacting or non-interacting. We follow the widely used evaluation measures including the classification accuracy (Acc), precision (Pre), sensitivity (Sn), specificity (Sp) and Matthews Correlation Coefficient (MCC) defined respectively as follows:

3 $${\text{Acc}} = \frac{{{\text{TP}} + {\text{TN}}}}{{{\text{TP}} + {\text{TN}} + {\text{FP}} + {\text{FN}}}}$$

4 $${\text{Pre}} = \frac{{{\text{TP}}}}{{{\text{TP}} + {\text{FP}}}}$$

5 $${\text{Sn}} = \frac{{{\text{TP}}}}{{{\text{TP}} + {\text{FN}}}}$$

6 $${\text{Sp}} = \frac{{{\text{TN}}}}{{{\text{TN}} + {\text{FP}}}}$$

7 $${\text{MCC}} = \frac{{{\text{TP}} \times {\text{TN}} - {\text{TP}} \times {\text{FN}}}}{{\sqrt {\left( {{\text{TP}} + {\text{FP}}} \right)\left( {{\text{TP}} + {\text{FN}}} \right)\left( {{\text{TN}} + {\text{FP}}} \right)\left( {{\text{TN}} + {\text{FN}}} \right)} }}$$

where TP, TN, FP, and FN represent true positive, true negative, false positive, and false negative, respectively.

## Data Availability

The source code of PRPI-SC and the used datasets are available at https://github.com/zhr818789/PRPI-SC. PlncRNADB dataset is downloaded from http://bis.zju.edu.cn/PlncRNADB/.

## Abbreviations

AE: Autoencoder
CD-HIT: Cluster Database at High Identity with Tolerance
CNN: Convolutional neural network
CTF: Conjoint triad feature
DAE: Denoising autoencoder
lncRNA: Long non-coding RNA
KH: K homology
RBP: RNA-binding protein
RF: Random forest
RPI: RNA protein interaction
RRM: RNA recognition motif
SAE: Stacked auto-encoder
SADE: Stacked denoising autoencoder
SGD: Stochastic gradient descent
SVM: Support vector machine
